# Working Memory Profiles in HIV-Exposed, Uninfected and HIV-Infected Children: A Comparison with Neurotypical Controls

**DOI:** 10.3389/fnhum.2017.00348

**Published:** 2017-07-06

**Authors:** Robyn Milligan, Kate Cockcroft

**Affiliations:** Department of Psychology, School of Human and Community Development, University of the WitwatersrandJohannesburg, South Africa

**Keywords:** HIV-exposure, HIV-infection, working memory

## Abstract

This study compared the working memory profiles of three groups of children, namely HIV-infected (HIV-I; *n* = 95), HIV-exposed, uninfected (HIV-EU; *n* = 86) and an HIV-unexposed, uninfected, (HIV-UU; *n* = 92) neurotypical control group. Working memory, an executive function, plays an important role in frontal lobe-controlled behaviors, such as motivation, planning, decision making, and social interaction, and is a strong predictor of academic success in school children. Memory impairments have been identified in HIV-I children, particularly in visuospatial processing. Verbal working memory has not been commonly investigated in this population, while it is unknown how the working memory profiles of HIV-EU children compare to their HIV-I and HIV-UU peers. Of interest was whether the working memory profiles of the HIV-EU children would be more similar to the HIV-I group or to the uninfected control group. The results revealed no significant differences in working memory performance between the HIV-I and HIV-EU groups. However, this does not mean that the etiology of the working memory deficits is the same in the two groups, as these groups showed important differences when compared to the control group. In comparison to the controls, the HIV-I group experienced difficulties with processing tasks irrespective of whether they drew on a verbal or visuospatial modality. This appears to stem from a generalized executive function deficit that also interferes with working memory. In the HIV-EU group, difficulties occurred with verbally based tasks, irrespective of whether they required storage or processing. For this group, the dual demands of complex processing and using a second language seem to result in demand exceeding capacity on verbal tasks. Both groups experienced the greatest difficulties with verbal processing tasks for these different reasons. Thus, disruption of different cognitive abilities could result in similar working memory profiles, as evidenced in this study. This has implications for the underlying developmental neurobiology of HIV-I and HIV-EU children, as well the choice of appropriate measures to assist affected children.

## Introduction

Working memory is a limited capacity system responsible for briefly holding and manipulating visuospatial and verbal material in a readily accessible form (Baddeley, 2000). It is regarded as a key executive function, together with cognitive flexibility and inhibitory control of automatized behaviors, and as such plays an important role in frontal lobe-controlled behaviors such as motivation, planning, decision making, and social interaction (Miyake et al., 2000; Miyake and Friedman, 2012). Measures of working memory are significant predictors of academic potential and success, even better than IQ scores (Berninger and Swanson, 1994; Swanson and Sachse-Lee, 2001; DeStefano and LeFevre, 2004; Swanson et al., 2004; Alloway and Alloway, 2013; Alloway and Copello, 2013; Alloway and Gregory, 2013). Furthermore, working memory assessments have reduced cultural and socioeconomic bias in comparison to conventional intelligence and scholastic measures, making them suitable for assessing learning in non-Western, low resource and developing communities (Engel et al., 2008; Rinderman et al., 2010; Cockcroft et al., 2016).

There is a growing body of research on the working memory functioning of neurotypical children. What is known within this population is that working memory is fractionated into several inter-related components, generally supporting the Baddeley (2000, 2012) model. Although several alternative working memory models exist (e.g., Ericsson and Kintsch, 1995; Cowan, 1999; Oberauer, 2010), Baddeley’s (2000) was selected as the theoretical basis for this study, as there appears to be the most theoretical consensus around this model, it is the most widely researched with pediatric populations, and it can be readily operationalized by psychometric measurement (Miyake and Shah, 1999). According to this model, working memory comprises three storage components, namely a visuospatial sketchpad (specialized for briefly holding and refreshing visual and spatial material), a phonological loop (responsible for auditory-verbal material), and an episodic buffer (responsible for integrating different types of material into meaningful episodes and communicating with long-term memory). These storage components are supervised by a flexible attentional controller, the central executive, responsible for controlled processing such as co-ordination of multiple tasks, temporary activation of long-term memory, maintaining task goals, and resolving interference during complex cognition (Baddeley et al., 1998a,b; Baddeley, 2000). The processing functions of the central executive may overlap with other executive control functions (Miyake and Friedman, 2012).

In neurotypical children, scores on working memory measures predict reading achievement, math ability, as well as learning and skill acquisition (Bull and Scerif, 2001; Swanson and Sachse-Lee, 2001; Cowan and Alloway, 2008; Alloway et al., 2009). Research into working memory functioning in neurodivergent populations includes children with Attention Deficit/Hyperactivity Disorder (AD/HD), Developmental Co-ordination Disorder (DCD), Specific Language Impairment (SLI), Autistic Spectrum Disorder (ASD), Down Syndrome, Williams Syndrome, Dyslexia, Dyscalculia, and general intellectual disabilities (Hughes et al., 1994; Bull and Johnston, 1997; McLean and Hitch, 1999; Passolunghi and Siegel, 2001, 2004; Swanson and Sachse-Lee, 2001; Laws and Bishop, 2003; Marton and Schwartz, 2003; Geurts et al., 2004; Pickering and Gathercole, 2004; Alloway and Gathercole, 2006; Pickering, 2006; Whitehouse et al., 2006; Williams et al., 2008; Alloway, 2011; Henry, 2012; Henry et al., 2012). There is general support for the fractionation of working memory following the Baddeley (2000, 2012) model, as well as evidence of distinct patterns of working memory deficits in these populations. For example, children with AD/HD show difficulties in central executive functioning, while those with DCD have deficits primarily in visuospatial working memory (Alloway and Gathercole, 2006; Alloway, 2011). Studies of children with SLI show that they have particular weaknesses in the phonological loop and central executive (Laws and Bishop, 2003; Marton and Schwartz, 2003; Pickering and Gathercole, 2004; Henry, 2012; Henry et al., 2012), while children with ASD experience greatest difficulty on central executive and visuospatial memory tasks (Hughes et al., 1994; Geurts et al., 2004; Whitehouse et al., 2006; Williams et al., 2008). Children with Dyslexia tend to be characterized by deficits in the phonological loop and central executive, while those with Dyscalculia also show central executive difficulties, as well as poor visuospatial working memory (Bull and Johnston, 1997; McLean and Hitch, 1999, Passolunghi and Siegel, 2001, 2004; Swanson and Sachse-Lee, 2001; Passolunghi et al., 2005; Passolunghi, 2006). These findings are useful as they enable theorizing about neurodivergent cognitive development in specific disorders, facilitate tracking the developmental progression of these disorders, and allow for appropriate remediation strategies to be implemented for affected children.

A population that is markedly absent from these studies is children with Human Immunodeficiency Virus (HIV) infection and exposure. Given the high incidence of pediatric HIV infection in Africa (World Health Organization [WHO], 2017), together with evidence that HIV-infected (HIV-I) children show an increased prevalence of learning difficulties (Sherr et al., 2009), as well as the importance of working memory for learning (Alloway et al., 2009), an investigation of working memory profiles in HIV-I and HIV-exposed but uninfected (HIV-EU) children is clearly warranted. An estimated 1,752,300 South African children between the ages of 5 and 14 years have reportedly been exposed to the virus *in utero* (Statistics South Africa, 2016), indicating a large group of children who are likely to be in need of specialized medical, neurocognitive, and educational assistance.

Most studies of neurocognition in HIV-I children have been generalist in nature [i.e., focusing on a general developmental or IQ score, obtained from the Griffiths Mental Development Scales (GMDS), the Bayley Scales of Infant Development, the Kaufmann Assessment Battery for Children (K-ABC), or the Wechsler Individual Scales for Children (WISC; Eley et al., 2008; Shead et al., 2010; Kandawasvika et al., 2011; Laughton et al., 2012, 2013; Lowick et al., 2012)]. Such studies may miss more subtle deficits within specific cognitive domains. A focused investigation of working memory profiles can provide detail on how this important executive function may be impacted by HIV infection and exposure. Of the few studies which investigated working memory, none appear to have measured all of its components (i.e., verbal and visuospatial storage, verbal and visuospatial processing), and most employed only a single measure, typically visuospatial in nature, thereby limiting construct validity. In most cases, the investigation of working memory was secondary to the investigation of another cognitive construct, such as executive functioning or general intelligence (Bagenda et al., 2006; Koekkoek et al., 2008; Allison et al., 2009). Our study addressed these shortcomings by using a detailed assessment comprising three measures of each component of working memory in order to compile comprehensive profiles of working memory functioning in three groups of children between 6 and 8 years matched for age, English-language ability, and socioeconomic status (SES). The three groups included HIV-I, HIV-EU, and HIV-unexposed, uninfected (HIV-UU) neurotypical children. Such profiles could assist with distinguishing the neurobiological mechanisms underlying HIV infection, and HIV exposure with no infection.

Working memory deficits have been identified in individuals infected with HIV, but these studies have generally focused on adults (Stout et al., 1995; Farinpour et al., 2000; York et al., 2001; Hinkin et al., 2002; Reger et al., 2002; Sacktor et al., 2002; Heaton et al., 2004; Dawes et al., 2008). It is difficult to reconcile the results of the few investigations of neurocognitive functioning in HIV-I children which include working memory measures due to sample differences in sociocultural and economic backgrounds (Lowick et al., 2012), different assessment measures, and very wide sample age ranges (Wachsler-Felder and Golden, 2002). Taking these difficulties into account, there is some evidence for the preservation of verbal storage (Blanchette et al., 2002; Bagenda et al., 2006; Martin et al., 2006; Klaas et al., 2009), and for impairments in visuospatial processing in HIV-I children (Boivin et al., 1995; Koekkoek et al., 2008). However, these studies had relatively small sample sizes (*N* = 14–41), and other than Bagenda et al. (2006), who included 6- to 12-year olds, all considered an older cohort of children (9+ years) than in the present study. None had a specific focus on working memory.

An important issue when investigating neurocognition in HIV-I children from Africa is the availability and adherence to antiretroviral treatment (ART), since only 28% of children in low and middle income countries who require ART actually receive it (Grantham-McGregor et al., 2007; Walker et al., 2011). The early initiation of ART appears to be key in preserving neurocognitive functions and possibly even reversing some of the damage caused by HIV infection, particularly in infants (Laughton et al., 2012). Similarly, there is evidence that HIV-I children who are not on ART show significant neurocognitive deficits relative to their uninfected counterparts (Shead et al., 2010). While ART plays a significant role in protecting neurocognition in HIV-I children, these children nonetheless struggle with neurocognitive deficits and delay relative to their uninfected peers (Smith et al., 2008; Cotton et al., 2009). This may be due to the neurotoxic effects of HIV infection which may cause permanent structural damage to the central nervous system before ART is initiated, and/or due to incomplete penetration of the blood–brain barrier by antiretroviral agents (Wolters et al., 1997; Van Rie et al., 2007; Cotton et al., 2009; Lowick et al., 2012). In our sample, all of the HIV-I children were on combination ART and had been virologically and immunologically stable for at least 6 months.

Due to improvements in preventative mother-to-child antiretroviral treatment (PMTCT) and its administration, the HIV-EU child population is rapidly overtaking the number of children born with HIV infection (Filteau, 2009; Shapiro and Lockman, 2010; Morden et al., 2016). The HIV-EU child is believed to have a unique neurocognitive profile due to exposure to the immunological side effects of HIV *in utero* (as a result of immune activation in the mother), as well as exposure to the prophylactic effects of PMTCT (Kuhn et al., 2001; Le Chenadec et al., 2003; Bunders et al., 2005; Nyoka, 2008; Garay and McAllister, 2010; Claudio et al., 2013). There has been limited research into the neurocognitive functioning of this population, and none that has explored how their working memory profiles may differ from those of children with HIV infection, or from uninfected controls. Investigations into the neurocognitive functioning of HIV-EU children have produced ambiguous findings. For example, there is some evidence that their neurocognition does not differ from that of neurotypical children (Kandawasvika et al., 2015), while other studies have found significant impairments in verbal functioning, sequencing, memory, and quantitative reasoning (Levenson et al., 1992; Brackis-Cott et al., 2009; Kerr et al., 2014). Kerr et al. (2014) compared the neurocognitive functioning of HIV-EU children from Thailand (*n* = 160) and Cambodia (*n* = 202) to an unexposed control group (*n* = 167). The groups were compared on the Child Behavior Checklist, the Beery Visual Motor Integration Test, the Stanford Binet-II and the Wechsler Preschool and Primary Scales of Intelligence, third edition (WPPSI-III). No significant group differences were found in executive function ability, but a significantly greater proportion of the HIV-EU sample had attentional difficulties. However, these results should be interpreted cautiously given the very wide age range of the sample (2–15 years). In another study, Kandawasvika et al. (2011) investigated the risk of HIV-associated neurocognitive impairment in 65 HIV-I infants who were part of a Zimbabwean PMTCT program in primary healthcare clinics. They were compared to 188 HIV-EU and 287 HIV-UU infants. A translated version of the Bayley Infant Neurodevelopmental Screener (BINS) was administered when the infants were 3, 6, 9, and 12 months old. This is a screening measure which identifies risk for developmental delay and neurological impairment in four areas, namely neurological functions, expressive functions, receptive functions, and cognitive processing. Infants were then classified according to their risk (low, moderate, or high), with 17% of those at high risk from the HIV-I group, 9% from the HIV-EU group, and 9% HIV-UU group (the remainder were of unknown status). The results showed that for this high risk group, the threat of neurocognitive impairment between the ages of 3 and 9 months increased from 3 to 6%, and this risk was highest among HIV-I infants (10% versus 2%: *p* < 0.001). The mothers of the HIV-I children tended to be older, more likely to be single, have no financial subsistence and to be co-infected with syphilis than the mothers of the other groups, factors which may have contributed to their infants’ underperformance. By 9 months, one-third of the uninfected infants were in the moderate risk group. These findings suggest that, even with PMTCT, there is risk of progressive encephalopathy for HIV-EU infants, and neurocognitive impairment may become increasingly more evident as these infants develop (Pollack et al., 1996).

Much of the research with HIV-I and HIV-EU children emerges from English-first language, Western, well-resourced contexts, and so has limited generalizability to children from sub-Saharan Africa, who experience a different, more virulent clade of HIV, and who are growing up in very different cultural, linguistic, and contextual circumstances (Chase et al., 2000; Wachsler-Felder and Golden, 2002; Laughton et al., 2013). This concern is also applicable to the studies that have profiled working memory functioning in other neurodevelopmental disorders, as well as in neurotypical children (Passolunghi et al., 2005; Alloway, 2011; Henry, 2012). The application of these findings to under-resourced, developing, African contexts where ART tends to be initiated later, is likely to be limited (Blanchette et al., 2002; Feinstein, 2003; Bagenda et al., 2006; Paxson and Schady, 2007; Smith et al., 2008). Importantly, samples from developing countries may be erroneously identified as showing significant neurocognitive delays if compared to Western norms in the absence of any matched neurotypical control group from the same socioeconomic, linguistic, and contextual backgrounds. For example, Lowick et al. (2012) compared the neurodevelopmental functioning of 30 HIV-I South African children on ART to that of 30 neurotypical controls (age range: 55–75 months). The HIV-I children had received combination ART for at least a year, and were virologically and immunologically stable. The standard scores from the GMDS-ER were used to categorize participants into developmentally delayed or not delayed groups. The HIV-I group had consistently higher proportions of children with developmental delay on all subscales of the GMDS-ER compared to the controls. Nearly half (46.7%) of the HIV-I group demonstrated severe developmental delay, compared to 10% of the neurotypical control group (*p* < 0.05); this reflects a sevenfold increase in the likelihood of severe neurodevelopmental delay in the HIV-I group (OR = 7.88; CI 1.96–31.68). Worryingly, 87% of the control group was classified as functioning in the below average to borderline range on the GMDS-ER, which may be a result of the context of extreme poverty and deprivation in which these children were growing up. The GMDS-ER is standardized on neurotypical British infants and children (Laher and Cockcroft, 2013). Similarly, Shead et al. (2010) found that the scores of their HIV uninfected group of infants (16–42 months) were more than one standard deviation below the age appropriate norms on the Bayley Scales. While these Scales have norms for South Africans (Richter and Griesel, 1988), they are outdated. This highlights the importance of using appropriate neurotypical comparison groups when sampling from non-Western, developing countries, and when using developmental measures that were normed in Western, developed and well-resourced contexts that tend to be culturally, socioeconomically, linguistically, and educationally different. Consequently, it was necessary in our profiling of the working memory functioning of HIV-I and HIV-EU children, to include a control group of neurotypical children from similar socioeconomic, linguistic, and cultural backgrounds, for comparison purposes.

The present study compared the working memory profiles of children who were HIV-I, HIV-EU, and an HIV-UU control group. As demonstrated in the review, working memory impairments have been identified in HIV-I children, particularly in visuospatial processing (Boivin et al., 1995; Koekkoek et al., 2008). It is unknown how the working memory profiles of HIV-EU children compare to their HIV-I and HIV-UU peers. Of interest was whether the working memory profiles of the HIV-EU children would be more similar to the HIV-I group or to the uninfected control group. We hypothesized that there would be significant differences in the working memory profiles of these groups, with the HIV-I group performing worst, and the HIV-UU group best.

## Materials and Methods

### Participants

All participants were African or Colored (mixed race), with an African language as their mother tongue and English as their second language. The HIV-I group comprised 95 children (49 girls; mean age = 7.42 years, *SD* = 0.85). All participants were in Grade 1 for the first time, attending English medium schools. We chose this age group as it marks the commencement of formal education in South Africa and is a valuable point at which to identify children who could benefit from additional educational assistance. Participants were drawn from public hospitals in Johannesburg, Gauteng. Inclusion criteria were an HIV positive status, on combination antiretroviral treatment (cART), viral suppression for at least 6 months (which includes adherence and a positive response to the cART, with no debilitating side effects from the medication, stable CD4 counts and viral loads). The HIV-EU group consisted of 86 children (47 girls, mean age = 7.36 years, *SD* = 0.88). These children were HIV negative, but their biological mother was HIV positive at the time of their birth. The participants received PMTCT at birth and subsequently seroconverted. The HIV-UU group comprised 92 children (55 girls; mean age = 7.05 years, *SD* = 0.86). Both they and their biological mother were HIV negative. Exclusion criteria for all groups were attendance at specialized schooling, institutionalization in an orphanage, or neurological compromise, such as epilepsy, traumatic brain injury, and/or previous diagnoses of Meningitis or Encephalitis.

### Measures

Each participant was individually assessed in a quiet room for one session of approximately 1 h. Measures were administered in a fixed order, designed to vary task demands and minimize participant fatigue. All tests were administered in English.

#### Working Memory

The 12 tests from the Automated Working Memory Assessment (AWMA; Alloway, 2007), an individual, computer based battery were administered. The AWMA comprises three tests of each component of Baddeley’s (2000) working memory model, namely Verbal (phonological loop) and Visuospatial (visuospatial sketchpad) Storage, Verbal and Visuospatial Processing (tapping central executive resources). It has a pre-determined sequence, is automatically scored and converted to a standard score based on the participant’s age *(M* = 100; *SD* = 15).

##### Verbal storage

These span tasks (Digit Recall, Word Recall, and Non-word Recall) measure the storage capacity of the phonological loop using different types of verbal material. In these tasks, either digits, words or non-words are stated in sequences that increase in number over each trial, starting with two items. The participant must recall them in the same order in which they were heard.

##### Verbal processing

Phonological loop and central executive functioning were measured in the complex span tasks of Listening Recall, Counting Recall, and Backward Digit Recall. In Listening Recall, participants judge the legitimacy of a spoken sentence by noting it as ‘true’ or ‘false,’ and must recall the final word of each sentence in sequence, after hearing a minimum of one and a maximum of six sentences. For Counting Recall, an array of shapes are presented, and participants must count and report the red circles, and then attempt to recall the total red circles for each array, in the original sequence. In Backward Digit Recall, participants must reverse the order of a sequence of heard digits, starting with two digits.

##### Visuospatial storage

Visuospatial sketchpad functioning (storage only) was measured with the Dot Matrix, Mazes Memory, and Block Recall tasks. In Dot Matrix, participants view four-by-four matrices and must identify the location of a previously shown red dot, by tapping on the correct square on the computer screen. For Mazes Memory, participants view a maze with a red pathway drawn through its course, and after a three second delay, must trace the path on a blank maze. In Block Recall, participants view a series of tapped blocks, and must reproduce the same sequence by tapping on each block on the screen.

##### Visuospatial processing

Visuospatial sketchpad and central executive functioning were measured with the Odd One Out, Mister X, and Spatial Recall tests. The Odd One Out test comprises three shapes, each presented in a row, and participants must detect the shape that is odd. At the end of each presentation (starting with one and reaching a maximum of six rows), participants must tap on the screen to recall the location of each odd-one-out shape in the correct order presented. In Mister X, pictures of two Mister X characters are shown, each wearing different colored hats, each holding a red ball, and each positioned in different orientations. Participants must identify whether the Mister X with the blue hat is holding the ball in the same hand as the Mister X with the yellow hat. At the end of six presentations, participants must recall, in the correct order, the position of each red ball by pointing to its location on the screen. The Spatial Recall test presents two objects (the target image has a red dot above it), and participants must identify whether the target object is identical or opposite of another presented object. The position of the red dot must be recalled at the end of each set of six presentations by pointing to its location on the screen.

Each test starts with a series of three practice trials, immediately followed by test trials, which progressively increase in difficulty. On practice trials, the correct response is given following the participant’s response, while no feedback is given on test trials. Each level offers six attempts, four of which have to be correct to proceed to the next level. Each level increases in difficulty with an added length to the item. Reliability and validity of the AWMA with British children are reported in Alloway et al. (2006, 2008). This test has not been standardized for South African children, but has been used in studies of South African children (Cockcroft and Alloway, 2012; Cockcroft, 2016; Cockcroft et al., 2016).

#### Non-verbal Intelligence

The Ravens Colored Progressive Matrices (RCPM; Raven et al., 1998) measured general intellectual ability to control for potential differences in this regard between the groups. The RCPM is a culturally reduced, non-verbal test consisting of 36 items in three sets, with 12 items per set. A single raw score is produced that can be converted to a percentile. The RCPM has good retest and split-half reliability, with no gender or ethnicity differences (Raven et al., 1990). Validity studies comparing the RCPM and WISC found strong correlations (0.91, 0.84, and 0.83) between the Full Scale, Verbal and Performance IQs, respectively (Martin and Wiechers, 1954).

#### English Language Proficiency

Since assessment was undertaken in English, which was not the mother tongue of the participants, the Sentence Repetition Test (SRT; Redmond, 2005) was used as a brief measure of English language proficiency in order to determine whether participants were proficient enough to complete the tests, and also to control for potential language differences between the groups. The SRT includes 16 ten-word sentences, each between 10 and 14 syllables long, with an even number of active and passive sentences. The participant must recall and repeat the sentences exactly as they are read and are scored either a 0, 1, or 2 based on their performance. The SRT was originally developed as a screen for children with Specific Language Impairment, however, it was subsequently found to be particularly sensitive to tapping the English proficiency of children who are not first language English speakers (Komeili et al., 2012; Komeili and Marshall, 2013). Inter-rater reliability was calculated by independent comparisons of marked responses (number of agreements/number of agreements + number of disagreements), and a value of 95% (sentence recall probe) and 98% (past tense elicitation probe) were found (Redmond, 2005).

#### Socioeconomic Status

The inclusion of a measure of SES was important due to its relationship with neurocognitive development and HIV infection and exposure (Coscia et al., 2001; Dobrova-Krol et al., 2010). The Living Standard Measure (LSM; South African Advertising Research Foundation [SAARF], 2012) is the industry standard when considering consumer patterns in South Africa. It is not dependent on reports of income or personal demographics (employment status, race, age, gender), and instead collects information about access to basic facilities, ownership of appliances and other assets, and factors related to residential location and type of dwelling in order to measure living standards as a proxy for SES. The measure is a 30 item binary questionnaire which marks the presence or absence of an appliance or facility in the family home (i.e., dishwasher, TV, mobile phone) as an indicator of wealth.

The study was approved by the Medical Research Ethics Committee of the University of the Witwatersrand, Johannesburg. Parents/guardians of participants provided informed, written consent, while participants granted assent to participate. There were appropriate opportunities for withdrawal at any point without prejudice.

## Results

### Descriptive Statistics

Skew and kurtosis for all of the variables met the criteria for univariate normality (Kline, 2005). The groups were balanced in terms of gender [HIV-I_(female)_ = 51.6%; HIV-EU_(female)_ = 53.41%; HIV-UU_(female)_ = 59.78], while there were significant between group differences on age [*F*(2,272) = 4.93; *p* = 0.008], SES [*F*(2,272) = 11.03; *p* < 0.001], English proficiency [*F*(2,272) = 31.29; *p* < 0.001] and intelligence [*F*(2,272) = 24.764; *p* < 0.001]. Descriptive statistics for the working memory, intelligence, English proficiency and SES measures are shown in **Table 1**, as well as analyses of variance between the three groups on these variables. Working memory composites reflect the average of the three tests that measure each of the following components: verbal storage, verbal processing, visuospatial storage and visuospatial processing.

**Table 1:** Descriptive statistics, ANCOVAs for working memory tests, ANOVAs for age, intelligence, English proficiency and SES by group.

Measure	HIV-I (*n* = 95)		HIV-EU (*n* = 86)		HIV-UU (*n* = 92)				
	Mean (*SD*)	Range	Mean (*SD*)	Range	Mean (*SD*)	Range	*F*	*p*	η^2^
Digit Recall	86.21 (14.11)	64–120	90.47 (17.00)	60–125	100.21 (17.05)	11–126			
Word Recall	77.54 (13.32)	30–127	76.15 (14.2)	63–120	94.43 (18.46)	63–129			
Non-word Recall	100.97 (17.87)	59–145	95.18 (17.15)	59–137	110.62 (18.03)	69–137			
**Verbal Storage**	85.6 (14.62)	59–131	84.44 (16.41)	59–129	102.41 (16.97)	69–129	11.29	0.000^∗^	0.078
Listening Recall	73.13 (15.31)	2–109	84.56 (17.58)	62–139	95.57 (16.89)	63–131			
Counting Recall	87.85 (13.65)	55–129	93.44 (13.74)	70–130	103.37 (20.36)	14–141			
Backward Digit Recall	80.57 (13.3)	58–119	84.56 (14.76)	58–136	99.42 (16.34)	64–143			
**Verbal Processing**	77.18 (11.13)	61–107	85.13 (14.13)	61–121	99.7 (16.26)	66–131	18.16	0.000^∗^	0.121
Dot Matrix	86.61 (16.72)	61–148	93.22 (14.71)	64–132	99.3 (17.17)	65–148			
Mazes Memory	83.51 (15.81)	48–133	86.94 (17.91)	48–129	97.09 (16.44)	59–133			
Block Recall	85.94 (13.57)	47–120	90.87 (13.02)	61–120	97.46 (16.08)	70–131			
**Visuospatial Storage**	82.76 (15.85)	2–131	88.74 (15.21)	63–126	97.29 (17.6)	63–139	2.42	0.091	0.018
Odd One Out	88.73 (17.53)	59–130	98.9 (15.93)	62–133	108 (17.36)	71–133			
Mister X	92.33 (15.92)	62–144	99 (13.62)	71–133	107.11 (19.42)	71–155			
Spatial Recall	87.65 (15.43)	60–126	95.56 (14.1)	64–135	102.32 (15.01)	70–135			
**Visuospatial Processing**	87.21 (16.38)	61–132	97.23 (14.14)	62–132	107.25 (17.98)	71–139	7.36	0.001^∗^	0.053
Age (months)	88.98 (10.15)	71–107	88.28 (10.51)	67–106	84.54 (10.35)	60–106	4.93	0.008^∗∗^	0.04
Intelligence	13.79 (5.09)	2–26	15.72 (6.01)	3–29	19.50 (5.79)	9–32	24.76	0.000^∗∗^	0.15
English proficiency	7.91 (6.95)	0–30	10.69 (7.46)	0–30	17.15 (9.83)	0–32	31.29	0.000^∗∗^	0.19
SES	6.55 (1.77)	2–10	5.88 (1.13)	2–10	6.95 (1.65)	2–10	11.02	0.001^∗∗^	0.08

*^∗^0.0125 correction for ANCOVAs between working memory tests after covariation of age, intelligence, English proficiency, and SES scores; ^∗∗^0.05 for all other ANOVAs.*

### Between Group Comparisons

A multivariate analysis of covariance (MANCOVA) between the working memory tests, with age, SES, language proficiency and intelligence as covariates, was significant for group [Wilk’s λ = 0.80, *F*(8,526) = 7.98, *p* < 0.0001, $\eta_{p}^{2}$ = 0.11, *power* = 1, Hotelling’s Trace = 0.244, *F*(8,524) = 7.85, *p* < 0.0001, $\eta_{p}^{2}$ = 0.11, *power* = 1]. Subsequent univariate analyses of covariance (ANCOVAs) indicated that significant between group differences were present in all composite scores except Visuospatial Storage [Verbal Storage *F*(2,270) = 11.28, *p* < 0.001, $\eta_{p}^{2}$= 0.078; Verbal Processing *F*(2,270) = 18.16, *p* < 0.0001, $\eta_{p}^{2}$= 0.12; Visuospatial Processing *F*(2,270) = 7.36, *p* < 0.001, $\eta_{p}^{2}$= 0.053; refer to **Table 1**]. The Bonferroni correction method was used to protect against an inflated familywise error rate, due to multiple comparisons, and therefore α was set at 0.0125.

Following from the ANCOVAs, pairwise comparisons (incorporating the same covariates mentioned above) highlight the differences between groups on the working memory composite scores (See **Table 2**). The Bonferroni correction for multiple comparisons (α = 0.0125) was used. The HIV-I group fared significantly poorer than the HIV-UU group on processing tasks (*p* < 0.001) irrespective of modality (verbal or visuospatial). The former group performed on a par with the neurotypical controls on the storage tasks across both modalities. In contrast, the HIV-EU group performed significantly worse than the HIV-UU group on tasks tapping the verbal modality, irrespective of whether their focus was on storage or processing (*p* < 0.001). The HIV-EU group’s performance was not significantly different from the neurotypical controls on measures drawing on the visuospatial domain, irrespective of whether these required storage or processing. The HIV-I and HIV-EU groups did not differ significantly from one another on any of the working memory composites.

**Table 2:** Pairwise comparisons between groups on the four working memory composites.

Working memory composites groups			Mean difference	*SE*	*p*	98.75% confidence interval	
						Lower bound	Upper bound
Verbal Storage	HIV-I	HIV-EU	4.356	2.165	0.136	–1.901	10.613
		HIV-UU	–6.874	2.541	0.022	–14.219	0.471
	HIV-EU	HIV-UU	–11.230	2.368	<0.001ˆ*	–18.073	–4.387
Verbal Processing	HIV-I	HIV-EU	–0.027	0.010	0.016	–0.055	0.001
		HIV-UU	–0.068	0.011	<0.001ˆ*	–0.101	–0.036
	HIV-EU	HIV-UU	–0.041	0.011	<0.001ˆ*	–0.072	–0.011
Visuospatial Storage	HIV-I	HIV-EU	–3.353	2.358	0.469	–10.170	3.463
		HIV-UU	–6.000	2.769	0.093	–14.002	2.003
	HIV-EU	HIV-UU	–2.647	2.579	0.917	–10.102	4.809
Visuospatial Processing	HIV-I	HIV-EU	–6.592	2.353	0.016	–13.393	0.209
		HIV-UU	–10.148	2.762	0.001ˆ*	–18.132	–2.164
	HIV-EU	HIV-UU	–3.556	2.573	0.505	–10.994	3.883

*^∗^*p* < 0.0125 (Bonferroni correction).*

### Within Group Comparisons

In order to determine the individual working memory profiles (relative strengths and weaknesses) for each group, repeated measures analyses of variance (rANOVA) were conducted between the four working memory composites within each group (see **Table 3**). For the HIV-I group, Verbal Processing was significantly weaker than the other three composites (Verbal Storage: *p* < 0.001, *d* = -0.65; Visuospatial Storage: *p* = 0.001, *d* = -0.41; Visuospatial Processing: *p* < 0.001, *d* = -0.72) with medium effect sizes. Visuospatial Processing was significantly stronger than Visuospatial Storage (*p* = 0.01, *d* = -0.28), but the effect size was small. For the HIV-EU group, *post hoc* analyses showed that Visuospatial Processing was significantly better than the other three composites (Verbal Storage: *p* < 0.001, *d* = 0.83; Verbal Processing: *p* < 0.001, *d* = 0.86; Visuospatial Storage: *p* < 0.001, *d* = 0.58), with medium to large effect sizes. The two verbal composites appear to be weaknesses as they have relatively lower scores, with no significant difference between the processing and storage components. For the HIV-UU group, Visuospatial Processing was significantly stronger than both Verbal Processing (*p* < 0.001, Cohen’s *d* = -0.44) and Visuospatial Storage (*p* < 0.001, Cohen’s *d* = 0.56), with medium effect sizes. Visuospatial Processing appears to be the strongest composite for this group, followed by Verbal Storage, Verbal Processing, and Visuospatial Storage in that order.

**Table 3:** Repeated measures ANOVAs comparing working memory performance within each group.

		HIV-I (*n* = 95)				HIV-EU (*n* = 86)				HIV-UU (*n* = 92)			
				98.75% CI				98.75% CI				98.75% CI	
		Mean difference	*p*	Lower bound	Upper bound	Mean difference	*p*	Lower bound	Upper bound	Mean difference	*p*	Lower bound	Upper bound
Verbal Storage	Verbal Processing	8.42	<0.001ˆ*	4.501	12.341	–0.69	0.999	–5.787	4.415	2.72	0.691	–2.7	8.135
	VS Storage	2.84	0.639	–2.681	8.359	–4.3	0.048	–9.339	0.735	5.12	0.014	–0.074	10.313
	VS Processing	–1.61	0.999	–6.629	3.408	–12.79	<0.001ˆ*	–17.974	–7.607	–4.84	0.035	–10.262	0.588
Verbal Processing	VS Storage	–5.58	0.001ˆ**	–10.066	–1.098	–3.61	0.142	–8.602	1.369	2.4	0.42	–1.754	6.558
	VS Processing	–10.03	<0.001ˆ*	–14.545	–5.518	–12.11	<0.001ˆ*	–16.704	–7.505	–7.55	<0.001ˆ*	–12.349	–2.76
VS Storage	Verbal Storage	–2.84	0.639	–8.359	2.681	4.3	0.048	–0.735	9.339	–5.12	0.014	–10.313	0.074
	Verbal Processing	5.58	0.001ˆ**	1.098	10.066	3.62	0.142	–1.369	8.602	–2.4	0.42	–6.558	1.754
	VS Processing	–4.45	0.01ˆ*	–8.789	–0.11	–8.49	<0.001ˆ*	–12.497	–4.479	–9.96	<0.001ˆ*	–14.08	–5.833
VS Processing	Verbal Storage	1.61	0.999	–3.408	6.629	12.79	<0.001ˆ*	7.607	17.974	4.84	0.035	–0.588	10.262
	Verbal Processing	10.03	<0.001ˆ*	5.518	14.545	12.11	<0.001ˆ*	7.505	16.704	7.55	<0.001ˆ*	2.76	12.349

*VS, visuospatial; CI, confidence interval; ^∗^*p* < 0.0125 (Bonferroni correction), ^∗∗^*p* < 0.001.*

In summary, while the HIV-EU and HIV-I groups’ performance did not differ significantly from one another on any of the working memory measures, when compared with the HIV-UU group, they revealed different areas of deficit. In particular, the HIV-I group showed difficulties with processing tasks irrespective of modality, while the HIV-EU group showed difficulties with verbal tasks, irrespective of whether they drew on storage of processing. When all the tasks were considered, both the HIV-I and HIV-EU groups showed the greatest relative difficulty with Verbal Processing.

## Discussion

The objective of this study was to investigate whether working memory performance differed between HIV-I children, HIV-EU children, and a group of neurotypical controls. While the overall results might suggest equivalent memory functioning between the HIV-I and HIV-EU groups, there were some important differences in memory functioning between them that emerged in the comparisons with the HIV-UU group. In comparison to the control group, the HIV-I group showed difficulties with processing tasks, regardless of whether they drew on a verbal or visuospatial modality. On the other hand, the HIV-EU group experienced difficulty with tasks that drew on the verbal modality, irrespective of whether they were storage or processing based. It is likely that these different working memory profiles stem from different etiologies.

Understanding these findings requires some detail about the measures used in this study. Tasks designed to tap working memory processing are complex, requiring simultaneous storage and processing of information, and thus draw on multiple cognitive functions including attention and long-term memory (Baddeley and Logie, 1999; Cowan, 1999; Duff and Logie, 2001). The complex memory span tasks used in the current study impose significant burdens on concurrent processing associated with the central executive (general working memory system), while the phonological and visuospatial storage/short-term memory tasks impose minimal processing loads, tapping instead the storage capacity of the respective working memory storage systems (Baddeley and Logie, 1999). Alternative theoretical accounts of complex working memory processing, where performance is supported by a unitary limited capacity resource that can support processing and storage in isolation or jointly, can also be explained by these tasks (Just and Carpenter, 1992; Cowan, 1999). Cognitive processing problems would therefore be expected to manifest themselves to a greater extent on the processing tests than the storage only tasks. This was evident when these tasks were compared within the HIV-I group; performance was significantly poorer on processing tasks relative to storage tasks.

The HIV-I children’s working memory profile (poor central executive processing) is similar to that of children with general learning difficulties and AD/HD (Bull and Scerif, 2001; Pickering and Gathercole, 2004; Alloway, 2011). Our finding of a processing impairment in the HIV-I sample is supported by evidence of impaired visuospatial working memory processing in HIV-I adult samples (York et al., 2001; Hinkin et al., 2002; Heaton et al., 2004; Dawes et al., 2008), and in two pediatric samples (Koekkoek et al., 2008; Boivin et al., 2010b). In our sample, we found that this processing impairment also impacted verbal working memory. Verbal working memory has not been generally investigated in other studies. This processing impairment may be linked to the diminished capacity of frontostriatal white matter networks, which are implicated in working memory processing and broader executive control (Van Rie et al., 2007). Since working memory shares integral links with other executive functions, particularly inhibitory control (Miyake and Friedman, 2012), it is most likely that the working memory impairment is secondary to a more general processing deficit, and is not in itself the cause of the difficulty. Due to these shared underlying processes, remediating working memory often has positive effects on broader executive functioning as well (Miyake and Friedman, 2012; Blakey and Carroll, 2015). In keeping with this line of reasoning, Hinkin et al. (2002) propose that working memory deficits across both verbal and visuospatial domains are a result of executive functioning impairment secondary to HIV infection, and not a result of localized damage of the more isolated cortical regions housing the verbal or spatial stores (left and right dorsal and ventral pathways; Van Rie et al., 2007; Makuuchi and Friederici, 2013). This argument is also supported by evidence that stimulant medication administered to treat comorbid AD/HD in HIV-I children improves both inhibitory control and working memory (Mehta et al., 2004). While such pharmacological treatments may benefit HIV-I children, behavioral interventions, such as working memory interventions, may also be of value, as these have demonstrated success with children with AD/HD (Klingberg, 2010). Impaired executive processes in the HIV-I group probably account for their considerably poorer performance with processing tasks.

In contrast, the HIV-EU group performed poorly on both storage and processing tasks that drew on the verbal modality. Their visuospatial processing and storage were on a par with that of the neurotypical controls. This does not suggest a general learning deficit, but rather a language-related difficulty (Pickering and Gathercole, 2004; Archibald and Gathercole, 2006; Pickering, 2006). Given that these children were assessed in their second language (English), and that they were in their first year of formal schooling in this language, it is most likely that the increased load of complex processing together with use of a second language meant that demand exceeded capacity in the verbal processing tasks for the HIV-EU group. There is evidence that the executive and attentional components of working memory are linked to long-term memory (e.g., Cowan, 1999; Oberauer et al., 2005). As such, the availability of language knowledge stored in long-term memory is likely to influence the efficiency of verbal working memory (Majerus, 2013). The capacity to process and store information, as captured in complex verbal working memory tasks, may be crucial to support learning generally (Pickering and Gathercole, 2004; Gathercole et al., 2006). This finding suggests that mother tongue instruction in the Foundation phase years would benefit these children and that too early a transition to second language instruction in English could be detrimental. It is important to note that the capacity of verbal working memory is likely to change as literacy development progresses (Conant et al., 1999; Conant et al., 2003; Boivin et al., 2010a). We did not measure early literacy skills, such as phonological awareness and alphabetic knowledge, in this study and so cannot comment on the extent to which the development (or lack thereof) of these skills may underpin this group’s weaker verbal working memory.

It is not possible to rule out general neurocognitive compromise in the context of HIV-exposure as an explanatory framework for the HIV-EU group. This may stem from the effects of ART toxicity and/or chronic maternal viral infection which disrupts neurogenesis in the fetus, and may result in permanent structural changes in the developing brain (Garay and McAllister, 2010). The fact that the HIV-EU group’s working memory performance generally fell in between that of the HIV-I and controls, suggests some depression of functioning in this group. Consequently, both the HIV-I and HIV-EU groups are likely to benefit from an integrated and targeted working memory intervention that focuses on strategies to promote working memory functioning, especially with regard to verbal processing.

A finding worth comment was the absence of impairment in visuospatial storage in both HIV-affected groups. Impairment in short-term visuospatial storage in HIV-I adults is usually characteristic of late stage infection, and is unlikely to show significant compromise during periods of good health (Reger et al., 2002). In addition, there is evidence for the dominance of visuospatial encoding in preschool children in keeping with the earlier maturation of the visual areas of the brain. This dominance begins to shift to a gradually increasing reliance on verbal encoding from approximately 6 years, the stage at which most children are exposed to literacy instruction (Hitch et al., 1988, 1993; Conant et al., 2003; Alloway et al., 2006; Boivin et al., 2010a). The finding that the visuospatial short-term stores in the two HIV-affected groups were no poorer than that of the control group could reflect this relative proficiency, as the participants were all school beginners who were just starting to negotiate this transition. This strength could be developed to compensate for poor verbal storage and processing capacities. Children with weaker verbal storage capacity may be able to capitalize on imagery or other kinds of visuospatial mediation to overcome some of their verbal learning difficulties. Strategic visuospatial mediation may be particularly valuable in mathematics (McLean and Hitch, 1999) and literacy (Johnston and Anderson, 1998), but less useful in the context of general language learning, as phonological forms are the basic representational medium (Pickering and Gathercole, 2004).

There are some limitations of the current study that warrant acknowledgment. Although children with formal diagnoses of AD/HD and learning disabilities were excluded from the study, it is possible that these difficulties may have been undiagnosed, particularly since the samples came from low SES circumstances with limited access to specialized educational or health care. Further, the effects of HIV infection and exposure without infection are accentuated by a host of socio-economic and psychosocial factors including additional illness, poor nutritional status, caregiver stress, and adverse living conditions (Walker et al., 2011). Studies where these risks were covaried (Floyd et al., 2007; Walker et al., 2011) found that the cognitive and motor deficits in HIV-EU children became non-significant. A socio-demographic analysis of the three groups in the current study indicated that the HIV-EU group were particularly disadvantaged in comparison to the other three groups. Their SES was the poorest and significantly lower than that of the HIV-UU group (*p* = 0.015); they had the highest familial burden of care (looking after an immediate family member with special needs) (21.95%), the highest proportion of recipients of a child support grant (91.9%), the lowest levels of maternal education (only 21.8% finishing high school), and the highest levels of paternal absenteeism (53.4%). In contrast, HIV-I children are frequently followed up on by specialized ART clinics which offer them support and access to social and allied therapeutic services, while the HIV-EU children seldom receive any of these auxiliary services, a finding replicated elsewhere (Kerr et al., 2014).

On the positive side, a strength is that this appears to be the first study to give a detailed comparative analysis of the working memory functioning of HIV-I and HIV-EU children. The participants were from poor socioeconomic backgrounds, and are representative of young children who access the public health system in South Africa. Thus, the results from this study should be generalizable to such a population where the incidence of HIV infection and exposure are highest, and which is most in need of support and intervention.

## Conclusion

There are widespread concerns about the early developmental wellbeing and loss of intellectual potential in South African children due to poverty, poor health and nutrition, and deprived environments (Grantham-McGregor et al., 2007). Of the reasons for this, lack of stimulation and HIV infection feature prominently (Walker et al., 2007). A key reason for the failure of HIV-I and HIV-EU children to attain their developmental potential could be because of poor working memory functioning since this would adversely affect their ability to mentally hold and manipulate information, skills vital for learning. Such failure would have far-reaching repercussions on long-term development and functioning. Early intervention at the level of working memory is clearly needed for HIV-affected children, and could have positive consequences for these children’s academic functioning, as well as their social and emotional efficacy.

## Author Contributions

KC helped to conceptualize this study, supervised the data collection and analyses, and wrote up the paper. RM helped to conceptualize this study, collected the data and analyzed it, and assisted with writing up the paper.

## Conflict of Interest Statement

The authors declare that the research was conducted in the absence of any commercial or financial relationships that could be construed as a potential conflict of interest.
